# DHA inhibits Gremlin-1-induced epithelial-to-mesenchymal transition via ERK suppression in human breast cancer cells

**DOI:** 10.1042/BSR20200164

**Published:** 2020-03-20

**Authors:** Nam Ji Sung, Na Hui Kim, Na Young Bae, Hyo Sik Jo, Sin-Aye Park

**Affiliations:** 1 Department of Medical Science, College of Medical Sciences, Soonchunhyang University, Asan-si, 31538, Republic of Korea; 2 Department of Biomedical Laboratory Science, College of Medical Sciences, Soonchunhyang University, Asan-si, 31538, Republic of Korea

**Keywords:** Breast cancer, DHA, EMT, extracellular signal-regulated kinases, GREM1

## Abstract

Docosahexaenoic acid (DHA) is an omega-3 fatty acid abundant in fish oils. It is known to have an inhibitory effect on various diseases such as inflammation, diabetes, and cancer. Epithelial-to-mesenchymal transition (EMT) is a process that epithelial cells gain migratory property to become mesenchymal cells involved in wound healing, organ fibrosis, and cancer progression. Gremlin-1 (GREM1) is a bone morphogenetic protein antagonist known to play a role in EMT. However, the role of GREM1 in the induction of EMT in human breast cancer cells and the effect of DHA on GREM1-induced EMT remain unclear. Establishment of GREM1 knockdown cell lines was performed using lentiviral shRNAs. Expression of EMT markers was determined by qRT-PCR and Western blotting. Effect of GREM1 and/or DHA on cell migration was investigated using wound healing assay. The level of GREM1 expression in human breast cancer tissues was determined by Oncomine database mining. GREM1 induced the expression of genes including N-cadherin, vimentin, and Slug. GREM1 promoted the migration of human breast cancer cells. GREM1 enhanced the expression of phosphorylated extracellular signal-regulated kinase (p-ERK) and the ERK activation was involved in EMT. Interestingly, DHA reduced the expression of GREM1. DHA also inhibited the expression of mesenchymal cell-associated genes and cell migration induced by GREM1. Furthermore, DHA suppressed the expression of p-ERK induced by GREM1. These results indicate that GREM1–ERK axis plays a role in EMT in human breast cancer cells and DHA is a putative compound that can inhibit EMT by inhibiting GREM1 signal transduction.

## Introduction

Breast cancer, one of the most frequent cancers in women worldwide, is known to have a high chance of cure if it is detected early. However, many patients experience chemotherapy resistance, tumor recurrence, and metastasis [1–3]. In addition, chemotherapy and/or hormonal suppression for breast cancer patients can cause serious side effects [4]. Since estrogen and estrogen receptors are major drivers in breast cancer progression, selective estrogen receptor modulators have been used to treat estrogen-dependent breast cancers [5,6]. HER2 protein is one of the most overexpressed receptors in breast cancer and is considered a biomarker involved in the treatment of HER2-positive breast cancer [7,8]. However, there is still a need for research and development of targeted therapies for triple negative cancers (lack of estrogen/ progesterone receptors and HER2) and other types of breast cancer patients [9,10]. In particular, the identification of molecular markers involved in the metastasis of breast cancer can be developed as an effective therapeutic target in breast cancer.

The epithelial-to-mesenchymal transition (EMT) phenomenon is a well-known process in which epithelial cells lose contact with each other and become mobile and invasive forms like mesenchymal cells [11]. Although EMT occurs normally during wound healing, excessive EMT induction is known to be involved in the fibrosis of various organs and tumor metastasis [12]. Many studies have shown that EMT is important for the metastasis of breast cancer [13,14]. EMT-related microRNAs can regulate EMT, invasion, and metastasis in breast cancer [15]. Twist, a key transcription factor for EMT, also promotes tumor metastasis in basal-like breast cancer [16]. In the same sense, breast tumor cell-specific knockout of Twist suppresses lung metastasis in mice [17].

Docosahexaenoic acid (DHA), a type of omega-3 fatty acid, is abundant in fish oils. DHA is known to possess diverse pharmacological properties including anti-inflammatory [18], anti-diabetic [19], and anti-carcinogenic effects [20]. In particular, DHA has been reported to suppress cancer progression by inhibiting EMT of cells. DHA prevents EMT and invasiveness of prostate [21] and colorectal [22] cancer cells promoted by transforming growth factor (TGF)-β-stimulated prostate fibroblasts or granzyme B, respectively. DHA inhibits vascular endothelial growth factor (VEGF)-induced migration of human umbilical vein endothelial cells [23]. It is also effective in inhibiting metastatic features of human cholangiocarcinoma cells via Twist suppression [24].

Gremlin-1 (GREM1) is one of the bone morphogenetic protein (BMP) antagonists that bind directly to BMP, followed by inhibition of the osteogenic action of BMP [25,26]. Grem1-deficient mice show an increase in bone formation [27], whereas Grem1-overexpressing mice exhibit a decrease in bone formation [28]. GREM1 is also involved in the fibrosis of various organs, including lung [29–31], kidney [32–34], and pancreas [35]. Interestingly, EMT phenomenon induced by GREM1 is known to be important for organ fibrosis [36] and tumor promotion [37,38]. GREM1 activates EMT process and profibrotic phenotype in tubular epithelial cells [36]. It has been reported that mesenchymal stromal cell-derived GREM1 can promote EMT in human esophageal squamous cell carcinoma [37]. Knockdown of GREM1 also inhibits EMT and invasion of glioma cells [38].

Likewise, GREM1 can stimulate fibrosis or EMT in various types of cells. However, the induction of EMT by GREM1 and its molecular mechanisms in human breast cancer cells remain unclear. In addition, it is not known whether DHA can inhibit GREM1-induced EMT. In the present study, we showed that GREM1 induces EMT and that extracellular signal-regulated kinase (ERK) activation by GREM1 is involved in the induction of EMT. Our results also showed that DHA suppresses GREM1-induced EMT by inhibiting GREM1 expression and/or GREM1-induced ERK activation.

## Materials and methods

### Cell culture and reagents

MDA-MB-453 and Hs578T cells were originally obtained from American Type Culture Collection. MDA-MB-453 cells were cultured in RPMI (Corning, NY, U.S.A.) containing 10% fetal bovine serum (FBS, Gibco, NY, U.S.A.) and 100 units/ml penicillin/streptomycin (Corning). Hs578T cells were cultured in DMEM (Corning, NY, U.S.A.) containing 10% FBS and 100 units/ml penicillin/streptomycin. Cells were maintained at 37°C in a humidified atmosphere with 5% CO_2_/ 95% air. DHA (purity > 98%) was purchased from Cayman Chemical Co. (Ann Arbor, MI, U.S.A.). Rabbit polyclonal GREM1 antibody was purchased from Abcam (Cambridge, U.K. Cat#. 140010). Recombinant human GREM1 was obtained from R&D systems (MN, U.S.A., Cat#. 5190-GR). Anti-N-cadherin (Cat#. 13116), anti-vimentin (Cat#. 5741), anti-Slug (Cat#. 9585), anti-pERK/ERK (Cat#. 8201), anti-pAkt/Akt (Cat#. 8200), and β-actin (Cat#. 4967) were obtained from Cell Signaling Technology (MA, U.S.A.). Recombinant human TGF-β (Cat #. 10804-HNAC) was purchased from Sino Biological Inc. (Beijing, China). U0126 (Cat#. 1144) was purchased from Tocris Bioscience (MN, U.S.A.).

### Gene silencing

TRC lentiviral GREM1 shRNA (TRCN0000063837) and shControl (shCtrl, SHC002) were obtained from Dharmacon (CO, U.S.A.). Lentiviruses were packaged in 293T cells. Cells were transiently transfected with shRNA vector together with pCMV-VSV-G and pCMV-dR8.91 using Lipofectamine 2000 (Life Technologies, MA, U.S.A. Cat#. 11668019). At 72-h post transfection, viral supernatant was collected, filtered, and used for transduction of breast cancer cells in the presence of 8 μg/ml polybrene (Merck Millipore, MA, U.S.A. Cat#. TR-1003-G). Stable cell lines were selected with 1 μg/ml puromycin (InvivoGen, CA, U.S.A. Cat#. ant-pr-1).

### Reverse transcription-quantitative polymerase chain reaction (RT-qPCR)

Total RNA was isolated from cells using TRIzol® (Invitrogen, CA, U.S.A. Cat#. 15596026). Reverse transcription of total RNA was performed using M-MLV reverse transcriptase (Promega, WI, U.S.A. Cat#. M1705) according to the procedure described previously [39] and to the provider’s protocol. qPCR was performed using qPCR kit (Nanohelix, South Korea, Cat#. QPCR-S500) and StepOnePlus Real-Time PCR (Thermo Scientific, MA, U.S.A.). RPL32 was used as the internal reference. Primer sequences are listed in Table 1.

**Table 1:** Sequences of primers used in qPCR

Target	Forward sequence	Reverse sequence
**CDH2**	CCACCTTAAAATCTGCAGGC	GTGCATGAAGGACAGCCTCT
**GREM1**	TCATCAACCGCTTCTGTTACG	GGCTGTAGTTCAGGGCAGTT
**VIM**	ATTCCACTTTGCGTTCAAGG	CTTCAGAGAGAGGAAGCCGA
**SNAI2**	GCAGTGAGGGCAAGAAAAAG	TCGGACCCACACATTACCTT
**RPL32**	TTAAGCGTAACTGGCGGAAAC	AAACATTGTGAGCGATCTCGG

### Western blot analysis

Standard sodium dodecyl sulfate polyacrylamide gel electrophoresis and Western blotting were used to analyze the expression of various proteins according to the procedure described previously [40]. Cells were homogenized, washed with ice-cold PBS, and lysed by cell lysis buffer (Cell Signaling Technology, Cat#. 9803) supplemented with complete EDTA-free protease and phosphatase inhibitors cocktails (Roche, CA, U.S.A.). The quantitative protein concentration was determined by BCA Protein Assay Kit (Thermo Scientific, MA, U.S.A.) and equal amounts of protein were loaded on 8–12% SDS-polyacrylamide gel electrophoresis. Proteins were transferred to polyvinylidene difluoride membrane (Merck Millipore, MA, U.S.A.) and subjected to immunoblotting using various antibodies overnight at 4°C followed by further incubation with the secondary antibody (AbFrontier, South Korea, Cat#. LF-SA8001 and LF-SA8002) at room temperature for 1 h. Visualization of protein bands was detected with Westsave Gold detection reagents (AbFrontier, Cat#. LF-QC0103).

### Wound healing assay

Cells were seeded into 6-well culture dishes and wounded by manual scratching of the surface with a 1 ml pipette tip. The scratched surface was washed with PBS to remove cell debris. Dishes containing these cells were then treated with the medium containing each compound and incubated at 37°C for 48 h. Phase contrast images of wound sites were captured at 0 h (control) and 48 h of incubation using an inverted microscope (4× magnification).

### Bioinformatics meta-analysis

To investigate the expression profile of GREM1 compared with TGF-β signaling system in human breast cancer versus normal breast tissue, we conducted a meta-analysis in the Oncomine database (http://www.oncomine.org) by setting the following search items: “GREM1”, “TGF beta signaling pathway”, “Breast Cancer vs. Normal Analysis”, and “mRNA”. All procedures were performed according to the instructions provided by Oncomine.

### Statistical analysis

Data are expressed as mean ± SD of results obtained from at least three independent experiments. Comparison between two groups was made using Student’s *t*-test. A *P*-value of less than 0.05 was considered to be statistically significant. *, *P* < 0.05; **, *P* < 0.01; and ***, *P* < 0.001.

## Results

### DHA inhibits TGF-β-induced EMT in human breast cancer cells

**Figure 1:**
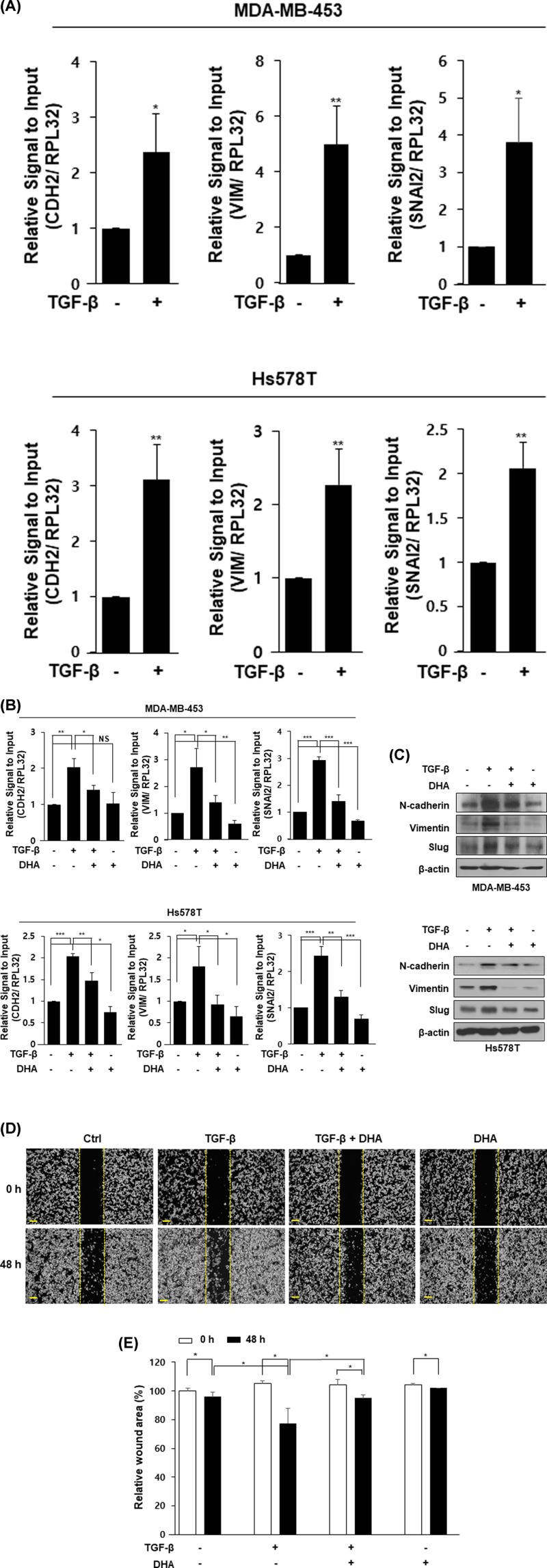
DHA inhibits TGF-β-induced EMT in human breast cancer cells Cells were starved overnight and stimulated with TGF-β (10 ng/ml) for an additional 48 h. RNA was analyzed by qPCR analysis (**A**). Cells were starved overnight and pretreated with TGF-β (10 ng/ml) for 24 h and then incubated with DHA (25 μM) for another 24 h. RNA was collected and analyzed by qPCR analysis (**B**) and proteins were performed by Western blot analysis (**C**). MDA-MB-453 cells were seeded in 6-well plates and wounded by 1 ml pipette tip. The cells were incubated with TGF-β (10 ng/ml) or DHA (25 μM) for 48 h, separately or in combination (**D** and **E**). *, *P*<0.05; **, *P*<0.01; ***, *P*<0.001, and NS=non-significant.

In the present study, we first examined whether DHA inhibits TGF-β-induced EMT in human breast cancer cells. As shown in Figure 1A, expression levels of genes (CDH2; N-cadherin, VIM; vimentin, SNAI2; Slug) involved in the mesenchymal cell phenotype were increased by TGF-β treatment in MDA-MB-453 and Hs578T cells. MDA-MB-453 and Hs578T cells were also treated with DHA in the absence or presence of TGF-β. Direct treatment with recombinant TGF-β protein increased mRNA (Figure 1B) and protein (Figure 1C) levels of N-cadherin, vimentin, and Slug. However, their levels were decreased by additional DHA treatment (Figure 1B,C). To determine whether this inhibitory effect of DHA on EMT contributes to cell migration, we next performed wound healing assay using MDA-MB-453 cells. TGF-β significantly increased cell migration whereas such increase was significantly inhibited by DHA treatment (Figure 1D,E). These results show that DHA has an inhibitory effect on EMT in human breast cancer cells.

### GREM1 is up-regulated in breast cancer tissues

**Figure 2:**
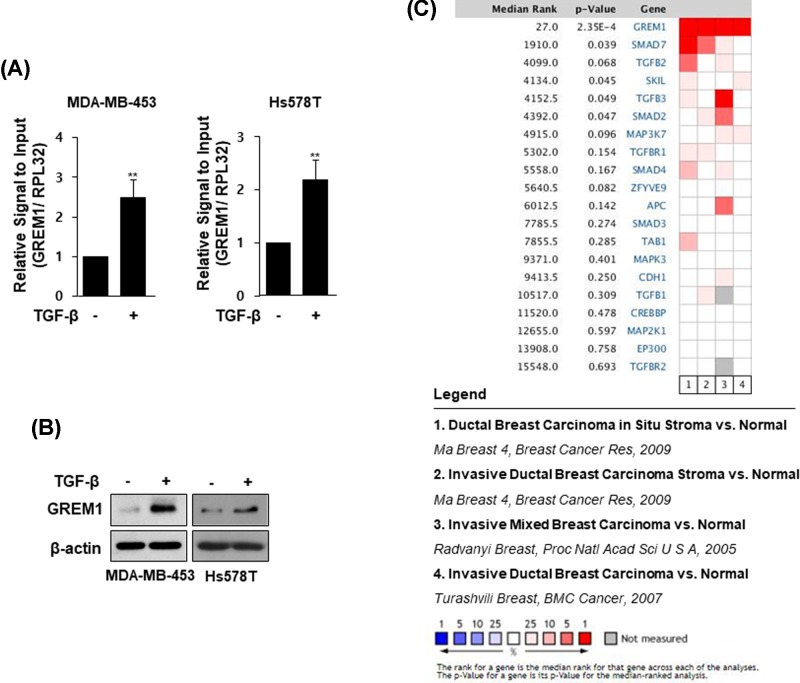
GREM1 is overexpressed in human breast cancer tissues Cells were starved overnight and stimulated with TGF-β (10 ng/ml) for an additional 48 h. RNA was analyzed by qPCR analysis (**A**) and protein lysates were subjected to immunoblot analysis (**B**). Oncomine microarray database was used to analyze GREM1 mRNA expression in breast cancer versus normal breast tissues. Four datasets were included in the meta-analysis (**C**); **, *P*< 0.01.

The levels of mRNA (Figure 2A) and protein (Figure 2B) of one of the EMT regulators, GREM1, were more increased by TGF-β treatment in MDA-MB-453 and Hs578T cells. We further investigated the level of GREM1 expression in human breast carcinoma tissues using Oncomine database. By adding a set of genes through the TGF-β signal pathway filter, the expression level of GREM1 could be compared with the levels of multiple genes corresponding to the TGF-β signal transduction pathway through four independent breast carcinoma versus normal analyses. The result showed that GREM1 is the most consistently highly expressed gene across breast carcinomas (Figure 2C).

### GREM1 induces EMT in human breast cancer cells

**Figure 3:**
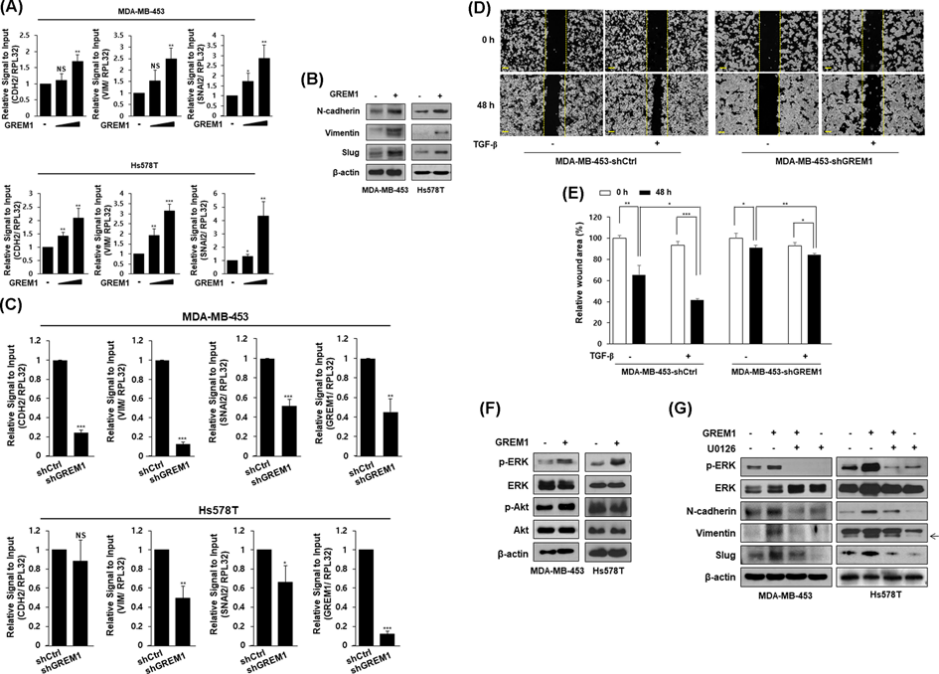
GREM1 increases EMT in human breast cancer cells Cells were starved overnight and stimulated with GREM1 (10 and 50 ng/ml) for an additional 48 h. RNA was analyzed by qPCR analysis (**A**). Cells were starved overnight and stimulated with GREM1 (50 ng/ml) for an additional 48 h. Protein lysates were subjected to immunoblot analysis (**B**). Each cell line was established by using lentiviral shRNA system. RNA was collected and indicated genes were quantitated by qPCR analysis (**C**). MDA-MB-453-shCtrl or MDA-MB-453-shGREM1 cells were seeded into 6-well plates and wounded with 1 ml pipette tip. Each cell line was incubated with vehicle or TGF-β (10 ng/ml) for 48 h. The wound closure was monitored by photography at the indicated time (**D** and **E**). Cells were starved overnight and treated with GREM1 (50 ng/ml) for 30 min. Protein lysates were subjected to immunoblot analysis (**F**). Cells were starved overnight and co-treated with GREM1 (50 ng/ml) and U0126 (10 μM) for 30 min (pERK and ERK) or 48 h (N-cadherin, vimentin, and Slug). Protein lysates were subjected to immunoblot analysis (**G**). *, *P*<0.05; **, *P*<0.01; ***, *P*<0.001, and NS = non-significant.

To further assess the effect of GREM1 on EMT in human breast cancer cells, MDA-MB-453 and Hs578T cells were treated with recombinant human GREM1 protein. Levels of mRNA (Figure 3A) and protein (Figure 3B) for mesenchymal cell markers including N-cadherin, vimentin, and Slug were increased by direct treatment with GREM1 protein. We established stable cell lines in which GREM1 expression was suppressed by a lentiviral shRNA system. Expressions of mesenchymal cell markers were significantly down-regulated in GREM1 knockdown cells (MDA-MB-453-shGREM1 and Hs578T-shGREM1) compared with those in each control cell line (Figure 3C). The expression of E-cadherin was also affected by GREM1 treatment or knockdown but the expressions of other EMT markers such as Twist and Snail were not significantly affected by GREM1 (data not shown). Moreover, TGF-β-induced cell migration was significantly suppressed in MDA-MB-453-shGREM1 cells (Figure 3D,E) compared with MDA-MB-453-shC cells.

Next, we examined whether GREM1 induces mitogen activated protein kinases (MAPKs) or protein kinase Akt in human breast cancer cells. As shown in Figure 3F, GREM1 protein treatment increased the phosphorylation of ERK, but not the phosphorylation of Akt. Interestingly, the increased expression levels of N-cadherin, vimentin, and Slug by GREM1 were reduced by treatment with U0126, an ERK kinase inhibitor (Figure 3G). These results strongly suggest that GREM1 plays an important role in the induction of EMT through ERK activation in human breast cancer cells.

### DHA inhibits GREM1 expression

**Figure 4:**
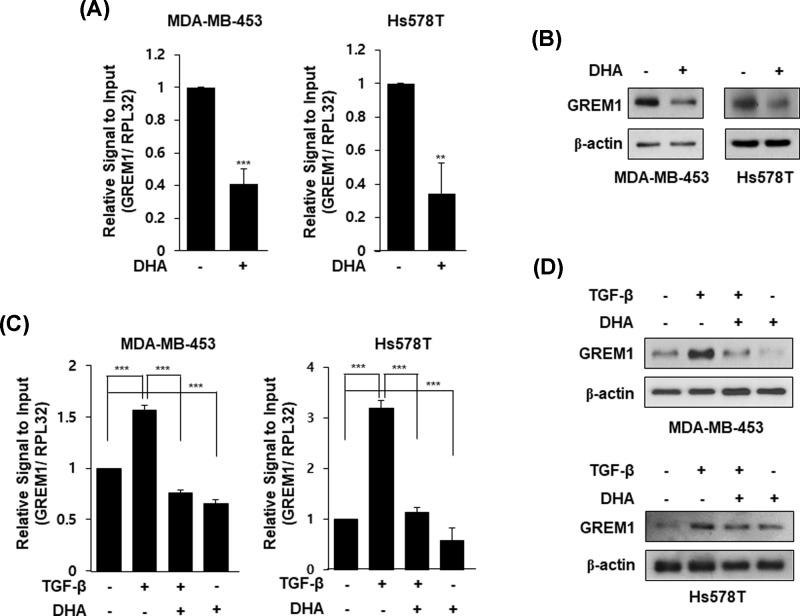
DHA inhibits GREM1 expression in human breast cancer cells Cells were starved overnight and stimulated with DHA (25 μM) for 24 h. RNA was collected and analyzed by qPCR analysis (**A**) and proteins were subjected to Western blot analysis (**B**). Cells were starved overnight and pretreated with TGF-β (10 ng/ml) for 24 h and then incubated with DHA (25 μM) for another 24 h. RNA was collected and analyzed by qPCR analysis (**C**) and proteins were subjected to Western blot analysis (**D**). **, *P*<0.01 and ***, *P*<0.001.

Although DHA has been proposed to inhibit EMT of cells, there have been no reports of whether DHA has an inhibitory effect on GREM expression. Therefore, we investigated whether DHA inhibits GREM1 expression in human breast cancer cells. Interestingly, mRNA level of GREM1 was suppressed by DHA treatment in MDA-MB-453 and Hs578T cells (Figure 4A). Its protein expression level was also reduced by DHA treatment in both cell lines (Figure 4B). In addition, TGF-β-induced GREM1 expression was decreased by DHA treatment (Figure 4C,D). These results suggest that DHA has an inhibitory effect on GREM1 expression in human breast cancer cells.

### DHA inhibits GREM1-induced EMT via ERK suppression

**Figure 5:**
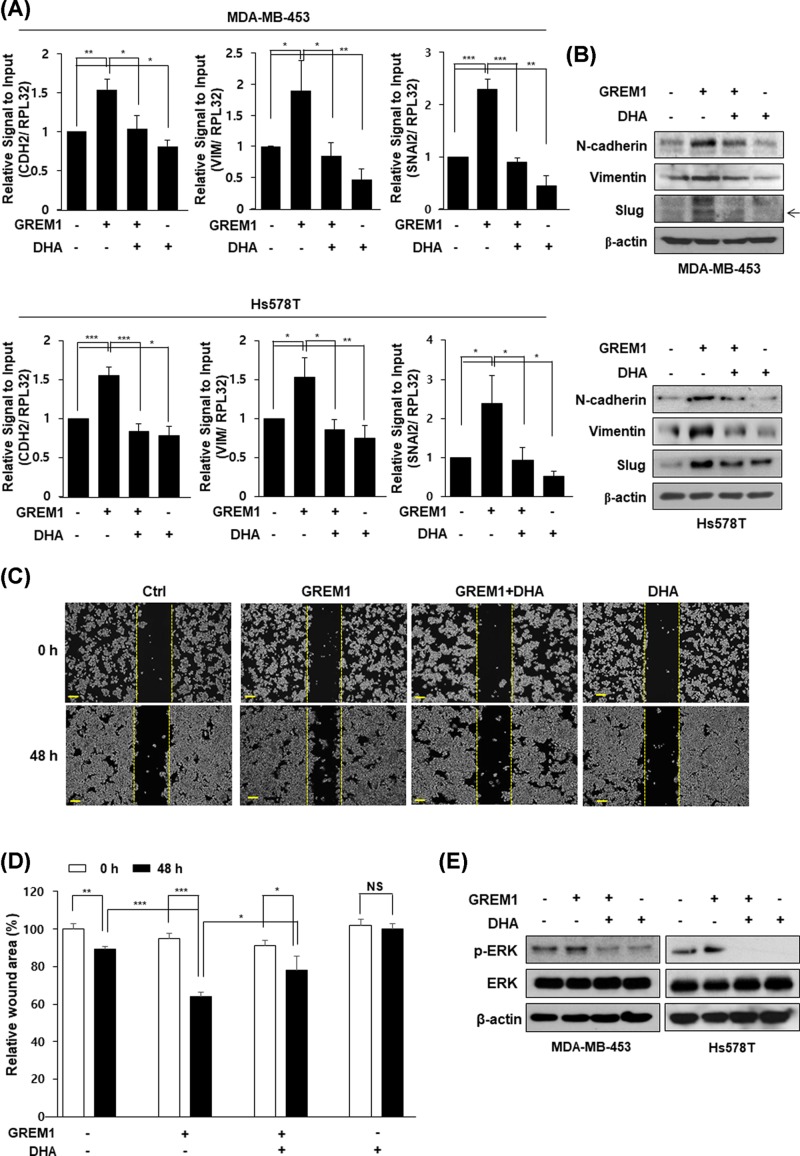
DHA inhibits GREM1-induced EMT Cells were starved overnight and pretreated with GREM1 (50 ng/ml) for 24 h and then incubated with DHA (25 μM) for another 24 h. RNA was collected and analyzed by qPCR analysis (**A**) and proteins were performed by Western blot analysis (**B**). MDA-MB-453 cells were seeded in 6-well plates and wounded by 1 ml pipette tip. The cells were incubated with GREM1 (50 ng/ml) or DHA (25 μM) for 48 h, separately or in combination (**C** and** D**). Cells were starved overnight and co-treated with GREM1 (50 ng/ml) and DHA (25 μM) for 30 min. Protein lysates were subjected to immunoblot analysis (**E**). *, *P*<0.05; **, *P*<0.01; ***, *P*<0.001, and NS=non-significant.

To further assess the effect of DHA on GREM1-induced EMT in human breast cancer cells, MDA-MB-453 and Hs578T cells were incubated with DHA in the absence or presence of human GREM1 protein. As shown in Figure 5A,B, direct treatment with GREM1 protein increased mRNA (Figure 5A) and protein (Figure 5B) levels of N-cadherin, vimentin, and Slug. Then, such increases were markedly suppressed by DHA treatment. Similar to TGF-β, direct GREM1 treatment also increased cell migration while such increase of cell migration was significantly inhibited by DHA treatment (Figure 5C,D). Furthermore, the increased level of phosphorylated ERK induced by GREM1 was significantly reduced by DHA treatment (Figure 5E). Taken together, these results suggest that DHA can inhibit GREM1-induced EMT by suppressing ERK activation in human breast cancer cells.

## Discussion

EMT is known to be involved not only in the metastasis of cancer cells, but also in the fibrosis of various organs [41]. The mRNA level of GREM1 is highly up-regulated in lung biopsies of patients with idiopathic pulmonary fibrosis [42]. Transient overexpression of GREM1 results in reversible fibrosis in rat lungs [30]. GREM1 contributes to fibrosis in other organs as well as in the lungs. GREM1 increases TGF-β production and activates the Smad signaling pathway in renal fibrosis [43]. In addition, GREM1 has been defined as a novel pro-fibrogenic factor in liver [44] and chronic pancreatitis [35]. Many studies have shown that GREM1 is involved in fibrosis of various organs, suggesting that GREM1 may be involved in EMT. In the present study, we examined a role of GREM1 as an EMT inducer in human breast cancer cells. GREM1 activated ERK signaling, which played an important role in GREM1-induced EMT in human breast cancer cells. Furthermore, we investigated the inhibitory effects of DHA on GREM1-induced EMT.

When EMT is induced, epithelial cells are transformed into mesenchymal cells, resulting in changes of various molecular markers. EMT-related transcription factors such as Snail, Slug, Smad, and Twist1 are known to regulate the expression of EMT markers. GREM1 activates EMT via the Smad pathway in cultured tubular epithelial cells [43]. GREM1 also activates Slug to increase EMT and the growth of mesothelioma cells [45]. GREM1-induced Slug expression is linked to the migration and invasive growth of mesothelioma cells [46]. In many cases, certain kinases, including ERK, c-Jun N-terminal kinase (JNK), p38, and Akt, regulate the activity of these EMT-related transcription factors. GREM1 increases hyperplasia and the invasiveness of synoviocytes via activation of ERK and Akt [47]. GREM1 activates the Akt signaling to promote proliferation, migration, and VEGF production in retinal pigmentation epithelial cells [48]. GREM1 utilizes both TGF-β/Smad and JNK/p38 MAPK signaling pathways to induce extracellular membrane proteins in human trabecular meshwork cells [49]. However, it has rarely been reported that increased specific kinase activity by GREM1 induces EMT of breast cancer cells.

Interestingly, GREM1 belongs to a protein group that has a cystine knot structural motif containing three disulfide bridges. Based on the fact that various membrane-receptor binding ligands, including growth factors, cytokines, and other immune modulators, have a common cystine knot structure, the cystine knot motif is known to be a characteristic of signaling molecules [50]. Therefore, GREM1 may act as a ligand for certain cell surface receptors and induce cell signaling involved in EMT, growth, migration, and invasion. It has been reported that GREM1 can directly bind to VEGFR2 [51]. The GREM1–VEGFR2 pathway is involved in renal inflammation [52] and the proliferation of retinal pigmentation epithelial cells [48]. In addition, GREM1 induces EMT via VEGFR2 activation in tubular epithelial cells [36]. Little is known about the binding between GREM1 and receptors, but further studies on the direct binding of GREM1 to specific receptors are needed to elucidate the mechanism of EMT induction.

It has been reported that DHA can inhibit EMT by suppressing the expression of α-smooth muscle actin in prostate fibroblasts [21]. Although DHA has been reported to be able to inhibit the metastasis of cancer cells [53–56], studies on molecular mechanisms by which DHA inhibits EMT are insufficient. Several studies have reported that BMP signaling can inhibit EMT. BMP-7 reverses TGF-β-induced EMT by increasing the expression of E-cadherin, a key epithelial cell adhesion molecule, in renal tubular epithelial cells [57]. BMP-7 inhibits aristolochic acid-induced myofibroblast phenotype and restores renal tubular epithelial morphology [58]. BMP-7 also attenuates the effect of TGF-β or silica on EMT in cholangiocarcinoma [59] or lung cancer cells [60], respectively. Therefore, further studies are needed to determine whether DHA can increase the expression of BMPs or modulate the activity of upstream signaling molecules, including transcription factors or kinases that regulate GREM1 expression.

Although many studies have reported the effects of natural products on inflammation and cancer, certain problems still need to be solved. Omega-3 fatty acids such as DHA have been widely used as therapeutic and nutritional supplements for animals. However, they have a variety of side effects in dogs and cats [61]. In addition, it has been reported that omega-3 fatty acids can reduce myogenesis while increasing adipogenesis in myotube formation, suggesting that maternal over-dosage of omega-3 fatty acids may affect fetal muscle development and intramuscular adipose tissue deposition [62]. The general mechanism for certain highly concentrated components of natural products or their safety in the human body has not yet been established in detail. Therefore, there is a need to provide precise guidelines for the mechanism, acceptable doses, and side effects of natural products.

## Conclusions

Our results showed that GREM1 is a new inducer of EMT in human breast cancer cells. GREM1 increased expression levels of mesenchymal cell-related proteins and ultimately enhanced the migration of breast cancer cells. In particular, GREM1 increased ERK activation, suggesting that the GREM1-ERK signaling might play a role in EMT of breast cancer cells. Furthermore, DHA had an inhibitory effect on the expression of GREM1 in human breast cancer cells. DHA inhibited the expression levels of mesenchymal cell-related proteins and cell migration induced by GREM1. Taken together, DHA has an inhibitory effect on EMT in breast cancer cells through suppressing GREM1 expression and/or GREM-induced ERK activation.

## Abbreviations

BMP: bone morphogenetic protein
DHA: docosahexaenoic acid
EMT: epithelial-to-mesenchymal transition
GREM1: gremlin-1
JNK: c-Jun N-terminal kinase
MAPK: mitogen activated protein kinase
p-ERK: phosphorylated extracellular signal-regulated kinase
TGF: transforming growth factor
VEGF: vascular endothelial growth factor
